# Crawling Creating Creatures On Beckett’s liminal minds

**DOI:** 10.1080/13825577.2015.1004916

**Published:** 2015-03-12

**Authors:** Marco Bernini

**Affiliations:** Department of English Studies, Durham University, Durham, UK

**Keywords:** Samuel Beckett, *Molloy*, *Malone Dies*, *The Unnamable*, *Company*, creature, cognition, phenomenology, consciousness, emergence, predictive mind, attunement

## Abstract

The continuity and contiguity between animal and human beings in Beckett’s work has been the subject of sustained critical attention. The recurring dehumanisation or degeneration of his characters’ mental faculties and behaviours has largely been analysed as an ‘ostensible animalization’ of human nature – following a reading of the ‘creaturely’ spectrum as a regression from the human to the animal. In contrast, this article considers the creaturely level in Beckett’s narrative as occupied by *undeveloped human cognisers* as opposed to (and sometimes rancorously opposing) fully fledged Humans. If Beckett’s formal minimalism has been extensively foregrounded, this essay draws on contemporary cognitive science and phenomenology in order to define and examine what the author calls Beckett’s *cognitive liminalism* – his literary exploration of liminal states of cognition and experience, of which the concept of the ‘creature’ constitutes a foundational element.

## Introduction

The continuity and contiguity between animal and human beings in Beckett’s work have been the subject of extensive critical attention (Bryden, 2013). The recurring dehumanisation or degeneration of his characters’ mental faculties and behaviours has been largely analysed as an ‘ostensible animalization’ (Weller, 2013: 18) of human nature – following a reading of the ‘creaturely’ spectrum as a regression from the human to the animal. As for the undermining outcome that this backward movement has for the idea of what human creatures are, Ulrika Maude (2013: 92) persuasively summarises the way in which ‘Beckett’s work casts doubt over all the major premises – consciousness, intentional subjectivity, and language – that have served to privilege the category of human’. By and large, existing approaches to Beckett’s portrayal of hampered human creatures rely on the categorial distinction between animals and humans in order to show how Beckett blurs the very divide between them, assimilating the latter to the former. In contrast, this essay aims to position Beckett’s creatures within a different segment of the creaturely scale: the portion ranging from lower levels of human cognition (human) to high-order degrees of human subjectivity (Human). In essence, in the human/Human gradation, I will consider the creaturely level in Beckett’s narrative as occupied by *undeveloped human cognisers* as opposed to (and sometimes rancorously opposing) fully fledged Humans or, as Beckett (2011: 86) writes in a letter to George Duthuit in 1948, ‘the illusion of the human and the fully realized’. The intention of the essay is not to sideline the importance of the non-human/human axis for the understanding of Beckett’s treatment of the creaturely realm, but rather to supplement the debate by pointing at gradations and conflicts *within* the human pole of the spectrum.

The main aim of the essay is therefore to provide a richer account of Beckett’s creatures in terms of their human (or not yet/no longer Human) cognitive faculties and of the phenomenological experiences they undergo in the developmental limbo in which they appear to be stuck. As we shall see, given the frequent occurrence of the term and its derivatives in his narrative work, the concept of the ‘creature’ suggests itself as a promising angle for an understanding of the model underlying Beckett’s fictional beings. In the first section, I briefly suggest how this creaturely model can be read as an integration of psychological, theological and narratological models of creatures. The following two sections concentrate on the specific cognitive (mis)functioning of these creaturely minds and on their peculiar phenomenological experience of the surrounding world. On the cognitive front, these central sections contrast contemporary scientific views on the mind’s emergent teleological nature (Deacon, 2012) and on the predictive power of the human mind (Hohwy, 2013) with the absence of these faculties in Beckett’s creatures. From the intertwined experiential perspective, they focus on the creatures’ bewildered perception of reality by drawing on the phenomenological notion of worldly ‘attunement’ (Ratcliffe, 2008). In the concluding section, I provide some interpretive hypotheses about the meaning of these feeble cognisers, to which I refer throughout the essay as *liminal minds*. If Beckett’s formal minimalism has been foregrounded early on (Brater, 1987), the ultimate aim of this essay is to define and to examine what I would label Beckett’s *cognitive liminalism* – his literary exploration of liminal states of cognition and experience, of which the concept of ‘creature’ constitutes a foundational element.

## Models of relations: creatures in psychology, theology and narratology

The term ‘creature’ tells first and foremost of a relation (with a creator and/or with other creatures), and it operates as such in Beckett’s narrative work. Models of creatureliness can be found in several disciplines, and the concept of the creature can be considered inherently interdisciplinary. Similarly, Beckett’s sources for modelling his creatures can be traced back to several areas of his interest as a reader (Nixon and Van Hulle, 2013). Without pretending to be exhaustive, the present section singles out three of the potential sources for Beckett’s creaturely modelling. The aim is to display how different models can overlap and be integrated with each other. Other models can be shown to underlie Beckett’s liminal creatures, yet there is enough evidence of Beckett’s interest in psychology (O’Hara, 1997), theology (Ackerley, 1999; Bailey, 2014) and narratological architectures to make these three models particularly significant. All three models provide a structure within which higher roles (occupied by the Super-Ego, God or the author) are put in relation with lower creatures (the Ego, worldly creatures, narrators and characters). All three models share a relational space in which creatures are located near to the lower boundary – a liminal border that Beckett’s creatures often attempt to cross in order to escape their relational fate.

Beginning with the psychological model, between 1933 and 1935, Samuel Beckett took extensive notes on psychology books (Feldman, 2006; see also Moorjani, 2004), notably on Freud’s ‘The Dissection of the Psychical Personality’ (1989), which is an explicit update of Freud’s 1923 essay on ‘The Ego and the Id’. In the earlier essay Freud (2001: 56) famously defined the Ego as a ‘poor creature owing service to three masters’ – the external world, the id and the Super-Ego. The term ‘creature’ disappears in his later account of the tripartite partition of the mind – the Ego here being described simply as the ‘poor ego’ (1989: 96) – as it did from Beckett’s meticulous summary of the Freudian mental structure (Engelberts and Frost, 2006). Yet the term was destined to become increasingly recurrent in Beckett’s work, from *Molloy* onwards, as a key term marking fictional beings (narrators, characters, narrating characters and other beings shaped through storytelling). Moreover, creatures in Beckett’s novels are also frequently qualified by the adjective ‘poor’ (a practice he also reproduced in his private correspondence; for instance, ‘poor old Molloy’; 2011: 240) or by semantic equivalents denoting a weakness in agency, consciousness and self-knowledge (Barry, 2008; Weller, 2013). In addition, the presence of several indefinite ‘masters’ who harass the poor fictional creatures (in *The Unnamable* alternatively defined as ‘my tormentors’, a ‘college of tyrants’ and so on; 2009: 341, 304) reinforces a remarkable resonance with Freud’s former study.

The resonance is not limited to semantic commonalities, but it also concerns the behaviour and position of the agents involved in the model. In fact, Freud’s model assigns to the Ego a submissive role, as it functions as a vicarious will obliged to conform to the authority of its masters. Moreover, this weakness in agency suggests a hierarchical structure between the ‘poor creature’ and its masters, a structure that if turned into a spatial model would locate the Ego at the bottom. In line with this mental spatialisation, the narrating figure in *The Unnamable* repeatedly defers the authority of his words to a mysterious master (‘I have spoken for my master, listened for the words of my master’; 2009: 304) and locates himself at the bottom (‘Perhaps after all I am simply in the basement’; 312) of a mental edifice (‘in my head, which I am beginning to locate to my satisfaction’; 344). At the lower level in which he is positioned – or into which he has fallen, like the rebellious Lucifer sunk into Hell in Dante’s *Divina Commedia* (Caselli, 2005) – the unnamable creature receives orders and information from his master, whose authority he suggests one should not inquire into, for in case he ‘turn[s] out to be a mere high official, we’d end up by needing God’ (2009: 368). For both Freud’s and Beckett’s creatures, their very existence is dependent upon, related to and bound by the masters they serve.

Moving to the theological domain, the last quotation from *The Unnamable* makes clear that the theological model is a sort of Ur-model for any creaturely hierarchy. Within it, God is located in a superior position as an overwhelming present absence, and this is why, as Mary Bryden (1998: 2) points out, ‘the hypothesised God who emerges from Beckett’s text is one who is both cursed for his perverse absence and cursed for his surveillant presence’. In the theological model of the world, in fact, God occupies the highest position, which is at the same time within *and* without the world itself. Creatures can only refer to God in worldly terms by what He is not, a negative description that Molloy appreciates when mirrored in the anthropological definition of man: ‘What I liked in anthropology was its inexhaustible faculty of negation, its relentless definition of man, as though he were no better than God, in terms of what he is not’ (2009: 35). As created creatures, humans have no access to the ontology and qualities of their creator; rather, they inhabit the same ontological basement as the Ego in Freud’s mental structure. However, and this marks an important difference between the psychological and the theological creaturely model, if the Ego cannot reproduce itself as other Egos upon which to act as master, humans *can* bring other creatures into their world, thus becoming themselves creators. In Beckett’s work, this human capacity to replicate themselves into other creatures generates what Paul Stewart (2011: 79) defines as a ‘horror for reproduction’ – a distaste that is not limited to parents, but also directed at their offspring, which are defined in *The Expelled* as ‘nasty little creatures’ (1995: 23).

This horror towards the creatures that decide, by becoming creators themselves, to emulate, in Malone’s words, ‘a sporting God to plague his creature’ (2009: 332) is equally present in Beckett’s narratological counterpart of the theological model. In classic narratological terms, the real flesh-and-blood author is never part of the fictional world he shapes; in Genette’s terms (1988), for instance, he is neither internal (*intradiegetic*) nor external (*extradiegetic*) to the fictional universe. The authorial level remains estranged from the suffering of the worlds and creatures it moulds, and for this reason it is always deprecated by Beckett’s fictional beings as a position occupied by a cruel ‘watch-maker’, as *Molloy* has it (2009: 31), who is exempt from the temporal ticking pain of existence (he is, for instance, defined as ‘the cankerous other’ in *Company*; 1996: 4).

In order to encounter the first proper creature *within* the worldly narratological architecture, we have to move from the boundary level of the author to the level of narration and to that of the characters. These two levels coincide (Walsh, 1997) whenever the narrator is to some extent a character inside/outside the storyworld or when the narrated characters tell stories themselves – and in so doing create an embedded narratorial (and creaturely) cascade. It is against this narrative (re)production of fictional beings that Beckett’s horror is often directed. In his narrative work, narrators are creatures who refuse to fulfil their reproductive role of shaping other beings through storytelling. This refusal is intended to cease the misery of (narrative) existence, as the anonymous narrator of the *Texts for Nothing* hopes: ‘That’s right, wordshit, bury me, avalanche, and let there be *no more talk of any creature*, nor of a world to leave, nor of a world to reach, in order to have done, with world, with creatures, with words, with misery, misery’ (1995: 137; emphasis added). In the narratological model, then, whenever the character is also a narrator, or vice versa, we find an in-between creature, synchronically created by the author and reluctantly authoring other character-creatures.

At the same time, Beckett’s narrators manifest a sort of narratological empathy towards the creatures they shape. This empathy is triggered by the fact that both narrators and characters share the same creaturely pain of being unwillingly brought into the world. Because of this ontological commonality, Beckett’s narrators see in their creatures a replication of their own suffering, a resemblance of their creaturely foundations, for which characters are therefore addressed by narrators as ‘fellow-creatures’ (‘mes semblables’; 2004: 18) as, for instance, in *The Unnamable* (2009: 292). To bring to an end the suffering of this (narrative) creaturely chain, we find Beckett’s narrators often engaging in a last round of creations in order to produce thereafter the final absence of creatures and worlds. For instance, at the beginning of *Molloy*, Molloy promises that ‘[t]his time, then once more I think, then perhaps a last time, then I think it’ll be over, with that world too. Premonition of the last but one but one’ (2009: 4). Similarly, in the second part of the same novel, Moran, as a narrator in charge of finding (that is, in a sense, creating) Molloy, promises that he ‘shall never light this lamp again’ (169) – the lamp being an image for the light of life. The relation to their characters is not the only narratological connection that Beckett’s narrating creatures try to sever with one last (de)creating manoeuvre. In *The Unnamable*, the narrating voice threatens his own masters (occupying the authorial God-level) with making them the object of his storytelling. In so doing, it would simultaneously subvert the ontological author–narrator relation and let the masters experience the qualitative feelings of creaturely pain:

they’ll know what it is to be a subject of conversation, I’ll impute words to them you wouldn’t throw to a dog, an ear, a mouth and in the middle a few rags of mind, … I’ll let down my trousers and shit stories on them, stories, photographs, records, lights, gods and fellow-creatures, … Be born, dear friends, be born, enter my arse, … They’ll see what it’s like, that it’s not so easy as it looks …

(2009: 373)

Within the narratological model, then, the narrating figure in *The Unnamable* represents a creature receding and regressing (Bernini, 2014) from his *created* ontology and his *creating* potentialities. This recession is to some extent present in many narrating creatures in Beckett’s work, and constitutes a key aspect for grasping the meaning of their liminal cognitive faculties and phenomenological experiences. Psychological, theological and narratological models of creaturely relation have provided important spatial and conceptual frameworks for modelling the liminal position of Beckett’s creatures. Oriented by this relational architecture, I will now describe in more detail the liminal features and principles operating (in) their peculiar minds.

## Liminal features: impeded logomotion and broken teleodynamics

In his 1929–30 lectures on *The Fundamental Concepts of Metaphysics* (*Die Grundbegriffe der Metaphysik*), Martin Heidegger famously proposed a distinction between objects, animals and humans according to the richness of their worlds (Kuperos, 2007). Material objects such as stones, Heidegger says, are ‘*worldless*’, animals are ‘*poor in world*’, whereas humans are ‘*world*-*forming*’ (1995: 177). By drawing on this distinction, Steven Connor (2014: 179) elegantly suggests that Beckett’s narrators hybridise the last two categories by acting as builders of poor worlds through narrative gestures of ‘miniature mundation’ – by creating and inhabiting impoverished little worlds (‘make a place, a little world’ as *The Unnamable*, quoted by Connor, has it; 2009: 398). Of the two features I want to present as defining Beckett’s liminal minds, the first resonates with Connor’s elaboration of Heidegger’s categories and is related to the developmental role of narratives in the navigation of the world. Heidegger explicitly assigns to language a key role in the exclusively human activity of world-forming. In Heidegger’s view, humans have a different openness towards the world that is made possible, as Kevin Aho (2007: 4) summarises, ‘by the fact that humans, unlike animals, dwell in *logos*’, where ‘*logos* is interpreted not as *ratio* but as language or, more specifically, as a linguistically structured space of meaning that allows beings to reveal themselves *as such*’. Language is in this sense a means of active exploration, which makes available and sets in motion our (re)cognition and navigation of the world. From a different, developmental angle, Katherine Nelson (2003: 19) explains the role of language acquisition and narrative performance in the emergence of consciousness by advancing an analogy with the mutual constructive relationship between visual perception and locomotion. Both in language and locomotion, Nelson explains,

each new level of understanding reached reveals new sources of knowledge and evokes a new effort at organization within and across domains … first sitting up, then crawling (new vistas appear), then cruising (again, new perspectives on old scenes and newly viewed objects), then walking freely (no longer tied to secure supports). The world was there all along, but the view from the infant’s eyes was **constrained by locomotor possibilities** and thus the sources of knowledge of the physical world were only gradually made accessible … But the existence of supporting structures in view provides the motivation for **boosting the self to the cruising level**, where such knowledge is accessible and where it can scaffold further excursions to new boundaries, which again makes new views and subsequent knowledge of the scene available.

(2003: 19; emphasis added)

As the development of our ability to stand and walk progressively increases our visual and informational fields, so language acquisition and narrative proficiency enable the navigation of new meanings and knowledge, thus making our cognitive and phenomenological experience of the world richer and more capacious. The functional analogy between language and locomotion is literally instantiated in Beckett’s liminal creatures, even if it operates negatively. Beckett’s characters are often completely (*Malone Dies*, *The Unnamable*, *Texts for Nothing*) or progressively (*Molloy*) reduced to a degree of immobility that parallels the impoverishment of their narrative control and understanding in a mutual deterioration that I would synthesise as *impeded logomotion*. For instance, Molloy, as Shane Weller (2013: 19) points out, undergoes a ‘lapse from the vertical to the horizontal’, first with the abandonment of ‘erect motion, that of a man’ (83), then taking to ‘crawling on his belly, like a reptile’ (84), and ending in immobility. With a similar degree of infirmity, Malone is writing in and narrating from a bed whose description recalls that of a hospital cot; and in *Company* we find an anonymous character ‘crawling and falling’ (1996: 35) in the informational darkness. In their impeded logomotion, the more mobility decreases, the more their narration becomes plagued by gaps, syntactical syncope and informational uncertainties – as Molloy notes: ‘when I say I said, etc., all I mean is that I knew confusedly things were so, without knowing exactly what it was all about’ (2009: 82). In the developmental view I am proposing, the interruption of the fertile co-evolution of logomotion and world-knowledge returns Beckett’s creatures to the underdeveloped crawling stage of infants, descending from the cruising level of boosted (Human) selves down into the limited accessibility to the world proper of (merely human) liminal creatures.

The second disruptive quality which characterises Beckett’s liminal creatures concerns the teleological disposition of the human mind. According to Terence Deacon (2012: 265), the ‘additional emergent transition’ of a fully fledged (Human) mind and consciousness is due to the ‘intrinsic incompleteness, an integral without-ness’ (2–3) in the organism. Human beings are always animated by desires, projects and thoughts that constitute, Deacon says, ‘absences’ towards which the mind and the organism are continuously directed – and our mind and experiences of the world ‘are shaped by and emerge from such specific absences’ (3). This incompleteness, then, ‘is a defining property of life and mind’ (3) that marks ‘the difference between a person and inanimate matter, between apparent freedom of thought and the rigid predictability of clockwork’ (55). In his theory of emergence, Deacon refers to the mind’s ‘end-directedness and consequence-organized features’ (265) (notably thoughts, the self and consciousness) as *teleodynamics*. It is this underlying teleological faculty of projecting plans, thoughts and mental representations into the future in order to modify or maintain present conditions that allows selves and consciousnesses to emerge, develop and evolve. Even emotions should be classed as teleodynamics features, Deacon (512) claims, as a sort of ‘infrastructure’ of every experience that allows the ‘tension that separates self from non-self: the way things are and the way they could be’ (512). Emotions function, therefore, as locomotors in so far as they push the organism further in time and space by generating a ‘perpetual becoming’, and for this reason Deacon proposes tweaking the term into ‘*e-motion*’ (512). Yet even this teleodynamic motility is negated or resisted by Beckett’s liminal creatures, following what I would therefore define as a *broken teleodynamics*.

Plans, purposes or e-motions through which self and consciousness emerge and develop are never internally generated or endorsed by Beckett’s creatures. When a feeble motivational drive enters the mind of Beckett’s creatures, it tends to originate in the authorial level in the form of a voice which addresses the creature with imperative commands. For instance, at the beginning of his journey, Molloy has no clear memory of why he has to go to his mother’s house: ‘My reasons? I had forgotten them. But I knew them, I must have known them, I had only to find them again and I would sweep, with *the clipped wings of necessity*, to my mother’ (2009: 23; emphasis added). Later on, the teleological necessity of his journey does not become clearer, and he starts to move in a circle within a forest, as a sign that the progressive linear locomotion of teleodynamics has, like Molloy’s body, been broken. Having no internal drive or e-motion to leave the forest, Molloy would happily stay there (‘I could have stayed there till I died, unripening, yes’; 80), enjoying the pleasure of completion – the fulfilment of simply being in the place you have to be, cancelling all teleodynamic tensions (‘being there I did not have to go there’; 80). Unfortunately, a higher-level order to move reaches Molloy from his ‘prompters’ (80), and suddenly he starts experiencing a feeling of incompletion:

But I could not stay there, stay in the forest I mean, I was not free to. That is to say I could have, physically, nothing could have been easier, but it was not purely physical, **I lacked something**, and would have had the feeling, if I had stayed in the forest, of going against an imperative …

(80; emphasis added)

In his own liminal mind, Molloy experiences no teleodynamic absences, yet a feeling of incompletion is instilled in him from superior narratological levels; some master wants him to emerge into mental life, to have consciousness (‘And then sometimes there arose within me, confusedly, a kind of consciousness’; 82) and to become Human.

A similar resistance to mental motion and e-motion can be found in Malone (‘I am satisfied, there, I have enough, I am repaid, I need nothing more’; 2009: 174), who struggles to remain ‘neutral and inert’, ‘tepid, without enthusiasm’ (173) while he waits to die stuck in his bed – the will to die being the ultimate subversion of the end-directed nature of Human mind and life. In *The Unnamable*, a broken teleodynamics also – and analogously – affects the drive to move (‘the essential is never to go anywhere, never to be anywhere’; 2009: 332); the liminal mind of the narrating creature recedes from and negates the whole worldly e-motional apparatus: ‘I never desired, never sought, never suffered, never partook in any of that, never knew what it was to have, things, adversaries, mind, senses’ (320). Finally, in *Company*, even the external voice – usually perturbing the liminal completion of the creatures with commands and desires – is responsible for the teleodynamic breakdown. In a sort of cruel narrative torture, a voice tempts the ‘one in the dark’ (1996: 3) to emerge into full Human existence by retelling his own memories (‘To one on his back in the dark a voice tells of a past’; 4). In so doing, the voice tries to *e-move* him away from his disremembered liminal state and ‘To confess, Yes I remember. Perhaps even to have a voice. To murmur, Yes I remember’ (10). However, this teleological temptation is cruelly put in the character’s mind only to dash any hopes for a future development of his present state (‘You will end as you now are’; 4).

Impeded logomotion and broken teleodynamics are, thus, two fundamental features of Beckett’s liminal minds – two core (dys)functional elements of their undeveloped situatedness in the world. As Michael Holquist (2002: 24, emphasis added) notes in his study on Mikhail Bakhtin, ‘the situatedness of the self is a multiple phenomenon: it has been given the task of *not* being merely given. It must stand out in existence because it is dominated by a “drive to meaning”.’ Language and narratives are fundamental elements that structure our sense of self in time and enable its evolving teleodynamics, its meaning-making incompleteness. This is why, as Holquist points out, ‘there is an intimate connection between the project of language and the project of selfhood: they both exist in order to mean’ (24), and meaning-making narratives are deeply integrated in our sense of being Human. This co-operation of language and selfhood, though, comes at the price of igniting the endless teleodynamic chain with its concomitant desires and (dis)illusions that generate a feeling of perpetual non-coincidence. In this respect, Bakhtin (1984: 59) describes Dostoevsky’s characters as an ‘infinite function’, as individuals tormented with the ‘profound consciousness of their own unfinalizability’. Conversely, Beckett’s creatures seek to *finalise* themselves – or, put in Beckett’s famous formula, to ‘fail better’ (1996: 89) in their narrative projects – by resisting the temptation of emerging into narrative existence (and of creating in turn narrative existences). They struggle to remain or to recede into the liminality that precedes narrative selfhood, identity, desires and e-motions. If the Human self is the outcome, as Gazzaniga (2009: 302) puts it, of a ‘knowledge structure’ responsible for ‘gluing together’ the ‘chaos of input’ into our autobiography, impeded logomotion and broken teleodynamics in Beckett’s cognisers overheat the glue of self, thereby dissolving the self back into a liminal unformed and experiential chaos. The resulting experience of the world these liminal minds undergo is thus full of informational noise and experiential uncertainty. In the next section, I will describe this cognitive bewilderment and phenomenological derailment using two principles (what I call *maximal prediction error* and *cognitive impenetrability*) which not only underlie the creation of Beckett’s liminal minds but, importantly, also affect the experience of reading his narrative worlds.

## Liminal principles: maximal prediction error and cognitive impenetrability

In his recent *The Predictive Mind* (2013), Jacob Hohwy elaborates a comprehensive account of cognition in terms of the predictive power of the mind. Hohwy maintains that our mind’s central faculty is the capacity to reduce errors in prediction by continuously updating its model of the world. The world, Hohwy (2013: 15) says, is ‘rife with regularities. Day follows night, seasons follow one another, more power corrupts, milk goes sour, faulty breaks are often followed by accidents’, and so on. By charting these regularities, our mind constructs models for interpreting the incoming perceptual stimuli, each time making top-down predictions and trying to minimise prediction error by continuously updating the model. Our encounter with the world is therefore guided by what Hohwy calls ‘perceptual inference’ (14), which is ‘a matter of hierarchical prediction error minimisation. Top-down predictions are compared to the actual sensory signal … and the difference, the prediction error, is used as a feedback signal for the internal models generating predictions’ (61). In a continuous comparison ‘between data and expectations about what the data should be, under a model of the world’, Human minds perfect their models in order to discard wrong hypotheses effortlessly, which are then ‘explained away’ (61). With Beckett’s liminal minds, however, cognition appears more costly: it seems rather to follow a principle of what I would call *maximal prediction error*.

Most of Beckett’s liminal minds are intent on incessantly making guesses about their perceptions and the worlds they end up in, but every hypothesis they produce is subsequently negated, undermined or impossible to verify. Beckett’s liminal minds are thus incapable of making reliable predictions and therefore of updating the model of the world they inhabit; because of that, they maximise the erroneousness of their predictions based on their perceptual inferences. This liminal cognitive condition in which the world is always resistant to predictions and presentiments is well exemplified by the following passage in *Molloy*:

But I can make no sense of this presentiment, and that I understand is very often the case with best presentiments, that you can make no sense of them. So perhaps it is a true presentiment, apt to be borne out. But can any more sense be made of false presentiments? I think so, yes, I think that all that is false may more readily be reduced, to notions clear and distinct, distinct from all other notions. But I may be wrong. But I was not given to presentiments, but to sentiments sweet and simple, to episentiments rather, if I may venture to say so.

(2009: 76)

Instead of charting regularities for the production of a model of the world increasingly *truer* (i.e. less inclined to prediction errors), Molloy says that the only signals he can pick out from the noise of the world are false presentiments. In other words, he can recognise incessant variances only against/without an attainable model of the world. This subversion of the predictive mind’s cognitive architecture leads to a sustained state of bottom-up unpredictable stimuli, where the sole regularity is perpetual variance – a principle that therefore continually maximises prediction errors (and, clearly, this principle in itself ‘may be wrong’). The examples of the liminal minds’ incapability of constructing a stable model of the world in Beckett’s work are endless, as is their attempt to reduce prediction errors. To mention just a few, Malone cannot guess who owns the room he is lying in (‘Present state. The room seems to be mine. I can find no other explanation to my being left in it’); and even when tentatively accepting the simplest perceptual inference, he nonetheless denies that this predictive strategy can cancel out the informational noise (‘It is better to adopt the simplest explanation, even if it is not simple, even if does not explain very much’; 2009: 176). Furthermore, the beginning of *The Unnamable* famously undermines the fundamental elements of perception (and of a storyworld) by questioning the identity of the place, the perceiver and the time of the narration (‘Where now? Who now? When now? Unquestioning. I, say I. Unbelieving’; 2009: 285). Finally, for the ‘one in the dark’ (1996: 3) in *Company* – who even struggles to assess whether the voice he hears is speaking to him or to another (‘If the voice is not speaking to him it must be speaking to another. So with what reason remains he reasons’; 6) – the surrounding world is an unpredictable informational noise in which ‘the greater part of what is said cannot be verified’ (3).

Past experiences and memory clearly play an important role in the predictive architecture of the mind. Perceptual inferences can be informed by our previous encounters with a similar worldly situation, according to a principle of ‘cognitive penetrability’ (Hohwy, 2013: 117–37). By contrast, given the frequent disremembered state of Beckett’s liminal minds, their limited faculty of making predictions is further weakened by a second principle that I would define as *cognitive impenetrability*. This impenetrability with regards to previously acquired information about the world in Beckett’s liminal minds is rooted in their faltering or lost memories of their past experience. As Gallagher and Zahavi (2008: 69) point out, ‘experiences never occur in isolation’, and it is the fact that our ‘past continually serves as the horizon and background of our present experience’ which makes us ‘able to navigate through a stream of experience without getting lost’. In our continuous ‘temporal navigation’ (69) of the world, previous situations are to some extent constantly *penetrating* our ongoing perceptions. In contrast, Beckett’s liminal creatures are always getting lost owing to having forgotten either their autobiographical information, as in *Malone Dies* (‘I had forgotten myself, lost myself’; 2009: 261), or the just-elapsed moment of their action, narrative performances included. Thus Malone frequently loses track of the stories he tells: ‘Then I forgot what I had said. A minimum of memory is indispensable, if one is to live really. Take his family, for example, I really know practically nothing about his family any more’ (201). The only informational exceptions that enlighten this cognitively ‘impenetrable darkness’ (2009: 77), as it is called in *Molloy*, are the memories and the worldly knowledge instilled by the voices the creatures hear from superior narratological levels. However, this authorial information is nonetheless questioned, resisted (as by the ‘one’ in *Company*) or barely retained, as in *The Unnamable*:

I remember little or nothing of these lectures. I cannot have understood a great deal. But I seem to have retained certain descriptions, in spite of myself. They gave me courses on love, on intelligence, most precious, most precious. They also taught me to count, and even to reason.

(2009: 292)

In terms of phenomenological experience (that is, of felt qualities that parallel but are not equal to our cognitive processes), the ability to predict future events and to access previous experiences is, like the teleodynamic projective nature of our mind, constitutive of our Human sense of self and of reality. In phenomenological terms, their correct functioning contributes to our ‘background sense of belonging to the world’ (Ratcliffe, 2008: 39–40). By drawing on Heidegger’s concept of ‘attunement’, Matthew Ratcliffe (2008: 8) describes our encounter with the world as a ‘realm of practical purposes, values, and goals … a background of practical significance’. However, in order for attunement to the world to count as a purposeful, meaningful navigation, we need to be able to perfect our predictions of its structure and to have a unified feeling of past experiences penetrating our present perception (the autobiographical sense of self being the enduring outcome of this temporal dynamic). With Beckett’s creatures, given their developmental resistance to acquiring new information or to retaining past experiences – a two-pronged temporal and cognitive withdrawal that makes impossible the very existence of a Human self – we are faced with an ‘altered sense of belonging’ (66). Beckett’s liminal minds appear to be before and/or beyond the emergence of the predictive machinery.

This undeveloped liminality also has important consequences for the experience of reading Beckett’s texts, as the same principles that keep Beckett’s creatures’ minds in a liminal state equally affect the reader’s habits in processing fictional Human minds. In fact, the two cognitive principles operating in Beckett’s liminal minds seriously challenge the reading disposition that Marie-Laure Ryan (1980: 406) has defined as a ‘principle of minimal departure’ which states that ‘we reconstrue the world of a fiction … as being the closest possible to the reality we know. This means that we will project upon the world of the statement everything we know about the real world, and that we will make only those adjustments which we cannot avoid.’ Analogously to what occurs in reality with perceptual inferences, in processing fictional worlds we compare our model of the world to the events we encounter, making predictions and subsequently updating the model (Kukkonen, 2014). In Eco’s image, readers perform ‘inferential walks’ (1984: 33, 214) outside the text (into the real world as well as into their previous cultural experiences of literary worlds) to access information that can help them optimise the model of the literary universe they are processing. However, as we have seen, it is precisely a stable and fully fledged model of the world that is missing from Beckett’s liminal minds – a cognitive quality that affects both creatures’ and readers’ predictions and mental projects. In conclusion, the cognitive liminality of Beckett’s fictional minds forces the readerly ‘principle of minimal departure’ continuously to fail because of the principle of maximal prediction error to which Beckett’s liminal beings are prey. Readers’ refreshing inferential walks thus become the same *inferential crawling* of Beckett’s creatures – stuck in a pre-Human developmental state in which the habitual percepts and the concepts of language narrative, time and selves are incapable of filtering the informational noise of the world from which Beckett’s creatures seek to recede even further.

## Conclusion: creatures for a geology of consciousness

The first prominent occurrence of the term ‘creature’ in Beckett’s work actually dates back to his 1931 essay on Proust. Here Beckett (1999: 11) explains how, in his view, Proust’s characters are ‘creatures’, victims of Time, which is a ‘monster of damnation and salvation’ that forces these earlier simple organisms into the (autobiographical, conceptual, extended, higher-order) Human mode of existence:

Proust’s creatures, then, are victims of this predominating condition and circumstance – Time; **victims as lower organisms**, conscious only of two dimensions and suddenly confronted with the mystery of height, are victims: victims and prisoners.

(1999: 12; emphasis added)

Once captured in Time, organisms are endowed (and burdened) with a sense of endurance and autobiographical consistency in which memory of the past ‘completes *the transformation of a creature of surface into a creature of depth* – unfathomable, accomplishes the solidification of a profile’ (50; emphasis added). Furthermore, the solidification of sensory experience into an autobiographical identity renders human beings, Beckett suggests, cognitively impermeable to the variant noises of reality; at the same time, he criticises a well-functioning memory as a ‘creature’ of the routine uniformity of (Human) experience:

The man with a good memory does not remember anything because he does not forget anything. His memory is uniform, **a creature of routine**, at once condition and function of his impeccable habit, an instrument of reference instead of an instrument of discovery.

(1999: 29–30; emphasis added)

In the same passage, Proust is accordingly praised as a man of ‘bad memory’ (29), for his narrative techniques manifest the gaps in memory and identity, thus partially liberating the creature within the Human from his (experiential, temporal, narrative) captivity.

Beckett’s own interpretation of Proust’s ‘creatures’, I think, provides an important ground for the understanding of what I have called Beckett’s *cognitive liminalism*. All the features and principles of Beckett’s creatures that I have presented might be seen as rendering liminal states of mind which precede (or oppose) the emergence of cognitive faculties that turn humans into Humans – creatures of sensory surface into creatures of conceptual depth. As Antonio Damasio (2000: 16) points out, human consciousness ‘is not a monolith’ and it can be divided into different levels. In Damasio’s terms, if the minimal level of (human) ‘*core consciousness*’ is a primary form of awareness of the surrounding world that animals and humans share, it is at the linguistic and conceptual level of (Human) ‘*extended consciousness*’ (16) that the complex autobiographical sense of self appears. Beckett’s ‘lower organisms’ are located on the edge of this extended level, struggling to resist becoming – or attempting to recede from being – creatures of time and memory, and perpetuating the pain of (narrative) existence by becoming creators themselves. Yet, as voiced in *The Unnamable*, these temptations are sometimes hard to resist (‘One may experience the need of such creatures’; 2009: 365) owing to the innate human ‘appetite for the imaginary’ (Gazzaniga, 2009: 220), of which the feeling and concept of a narrative self (Schechtman, 2011) are the most familiar results. As Beckett (2011: 102) writes in another letter to Duthuit in 1948, his worlds and creatures are located ‘in the eternally larval’, attempting to render ‘the courage of imperfection of not-being too, in which we are intermittently assailed by the temptation still to be’. In his ‘Whoroscope’ Notebook, together with his drawing of a table of geological eras, Beckett alludes to a possible ‘geology of conscience’ (where the word is used in the French meaning of consciousness; see Nixon and Van Hulle, 2013: 210–12). This essay has aimed to show how Beckett executes this plan by going beyond the strata of Human extended consciousness, back to the limen in which Beckett’s beings are *crawling away* from the ontological prison of becoming creating created creatures.
